# Role of gut microbiota-derived signals in the regulation of gastrointestinal motility

**DOI:** 10.3389/fmed.2022.961703

**Published:** 2022-07-22

**Authors:** Zhipeng Zheng, Jingyi Tang, Yingnan Hu, Wei Zhang

**Affiliations:** Department of General Surgery, The Second Affiliated Hospital of Zhejiang Chinese Medical University, Hangzhou, China

**Keywords:** gut microbiota, gastrointestinal motility, gut microbial components, bile acid, short-chain fatty acids (SCFAs), tryptophan metabolites

## Abstract

The gastrointestinal (GI) tract harbors trillions of commensal microbes, called the gut microbiota, which plays a significant role in the regulation of GI physiology, particularly GI motility. The GI tract expresses an array of receptors, such as toll-like receptors (TLRs), G-protein coupled receptors, aryl hydrocarbon receptor (AhR), and ligand-gated ion channels, that sense different gut microbiota-derived bioactive substances. Specifically, microbial cell wall components and metabolites, including lipopeptides, peptidoglycan, lipopolysaccharides (LPS), bile acids (BAs), short-chain fatty acids (SCFAs), and tryptophan metabolites, mediate the effect of gut microbiota on GI motility through their close interactions with the enteroendocrine system, enteric nervous system, intestinal smooth muscle, and immune system. In turn, GI motility affects the colonization within the gut microbiota. However, the mechanisms by which gut microbiota interacts with GI motility remain to be elucidated. Deciphering the underlying mechanisms is greatly important for the prevention or treatment of GI dysmotility, which is a complication associated with many GI diseases, such as irritable bowel syndrome (IBS) and constipation. In this perspective, we overview the current knowledge on the role of gut microbiota and its metabolites in the regulation of GI motility, highlighting the potential mechanisms, in an attempt to provide valuable clues for the development of gut microbiota-dependent therapy to improve GI motility.

## Introduction

The fundamental gastrointestinal (GI) functions include motility, sensation, digestion, absorption, secretion, and barrier function. Among these functions, the primary responsibility of GI motility is to mix gut contents with digestive secretions and expose them to the absorptive surface, to accomplish propulsion along the GI tract, to prevent retrograde movement of contents, and to dispose of residues, which is essential for orderly digestion of food, appropriate absorption of nutrients and timely expulsion of unwanted wastes (1). A better understanding of GI motility is important for the prevention and treatment of GI disorders, such as irritable bowel syndrome (IBS), functional constipation, and post-operative ileus (POI). IBS is a functional GI disorder with symptoms including abdominal pain and a change in stool form or frequency, which affects around 1 in 10 people globally with a wide variation of prevalence in different regions (2). Genetics, diet, and the gut microbiota are recognized risk factors for IBS and the pathophysiology includes GI motility disturbances, visceral hypersensitivity, and altered central nervous system (CNS) processing (2, 3). The main treatment of IBS includes patient education about dietary changes and antispasmodic drugs, but people with severe symptoms may also need central neuromodulators, intestinal secretagogues, drugs acting on opioid or 5-hydroxytryptamine (5-HT) receptors, antibiotics, and psychological therapies (3). Functional constipation has a prevalence of 14% in adults with common pathophysiological factors including genetic factors, lifestyle factors, and psychological disorders (4). Management of functional constipation is dependent on different subtypes: normal transit, slow transit, or an evacuation disorder, involving lifestyle interventions, pelvic floor interventions, and pharmacological therapy (4). POI is a common clinical problem that complicates the recovery of up to 30% of patients undergoing GI surgery (5). These different GI disorders undergo a similar pathophysiological process in which GI motility is disturbed.

**FIGURE 1:**
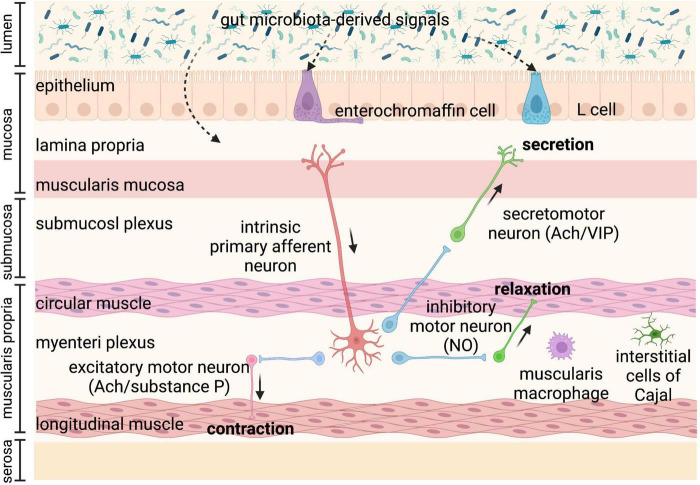
Anatomy of the bowel wall ensures the effect of gut microbiota on gastrointestinal (GI) motility. Gut microbiota is geographically close to the bowel wall, which is composed of the mucosa layer (epithelium, lamina propria, and muscularis mucosa), the submucosa layer (submucosal plexus), the muscularis propria (circular smooth muscle, myenteric plexus, and longitudinal smooth muscle), and the serosa layer. Enteroendocrine cells (enterochromaffin cells and L cells) dispersed among the mucosa layer can directly sense gut microbiota-derived signals and then secrete hormones, such as glucagon-like peptides (GLPs) and peptide YY (PYY) (L cells), and serotonin (enterochromaffin cells), affecting enteric nervous system (ENS) and gastrointestinal (GI) motility. The ENS comprising submucosal plexus and myenteric plexus plays a central role in GI motility and can also sense and respond to gut microbiota-derived stimuli that cross the epithelium. The myenteric plexus is responsible for the propulsion of intestinal contents under the movement of the smooth muscle, while the submucosal plexus is mainly involved in the secretion and absorption. Intrinsic primary afferent neurons (IPANs) are activated by gut-derived signals and activate ascending and descending interneurons, which stimulate inhibitory and excitatory motor neurons, as well as secretomotor neurons. Besides, musculari macrophage and interstitial cells of Cajal (ICCs) in muscularis propria can be activated by gut microbiota-derived signals affecting GI motility. Ach, acetylcholine; NO, nitric oxide; VIP, vasoactive intestinal peptide.

Gastrointestinal motility is regulated by the coordination of various factors, including the enteric nervous system (ENS), immune system, gut hormones, as well as gut microbiota (6–8). Gut microbiota-regulated GI motility is based on the unique architecture of the GI tract (Figure 1). The bowel wall is composed of the mucosa layer (epithelium, lamina propria, and muscularis mucosa), the submucosa layer (submucosal plexus), the muscularis propria (circular smooth muscle, myenteric plexus, and longitudinal smooth muscle), and the serosa layer (8). Enteroendocrine cells (EECs) dispersed among the mucosa layer of the GI tract are key players in the communication between the gut microbiota, the ENS, and the GI motility, through producing and secreting a variety of hormones or signaling molecules, such as glucagon-like peptides (GLPs) and peptide YY (PYY) (L cells), and serotonin (enterochromaffin cells) (9). EECs, also termed “neuropod cells,” directly communicate with neurons through modified synapses (10). Enterochromaffin (EC) cells are responsible for the major production of 5-HT, which functions as a critical activator of many GI reflexes by signaling through a variety of receptors located on the ENS (11). The ENS comprises a large number of neurons, and the majority of them are in the submucosal plexus and myenteric plexus. Profiling of the ENS at single-cell resolution has been used to identify colonic neuronal types: (1) sensory neurons, also called intrinsic primary afferent neurons (IPANs), which sense and respond to chemical and mechanical stimuli; (2) interneurons, which transfer signals between neurons; (3) secretomotor neurons, which induce secretions in other cell types and control blood flow; (4) excitatory motor neurons and (5) inhibitory motor neurons, which together innervate longitudinal and circular smooth muscles and coordinate muscle contraction and relaxation in the GI tract (12). The myenteric plexus is responsible for the propulsion of intestinal contents under the movement of the smooth muscle, while the submucosal plexus is mainly involved in the secretion and absorption (8, 13). In particular, motor neurons innervate circular muscle consisting of two main types of functionally distinct myenteric neurons: ascending excitatory neurons, containing neuromediators or enzymes such as choline acetyltransferase (ChAT) and substance P; and descending inhibitory neurons, containing vasoactive intestinal peptides (VIPs) and neuronal nitric oxide synthase (nNOS) (14). Non-neuronal, the interstitial cells of Cajal (ICCs) are pacemaker cells located in the same area as the myenteric plexus and are important for phasic myogenic contractions by the generation of electrical oscillatory activity (15). ICCs are responsible for peristalsis, and the electrical signaling of ICCs underlying rhythmic muscle contractions is most relevant to the segmentation motor patterns of the GI tract (16). Besides, a distinct population of macrophages is distributed in the muscularis propria of the GI tract, called muscularis macrophages (MMs), taking part in the regulation of colonic peristaltic activity (17).

The sensory components in the gut wall can detect luminal substances from gut microbiota, which directly or indirectly modulate GI motility. Certain gut microbial substances bind to receptors on the luminal cell layer of the mucosa, such as enterochromaffin cells (ECs) and L cells, and initiate downstream signals that can activate receptors on enteric neurons to regulate GI motility (18). In addition, 90% of 5-HT in the intestine is produced by ECs, and 5-HT secretion is thought to be important in regulating GI motility (19). Alternatively, some microbial cell wall components or metabolites first need to cross the intestinal epithelial cell layer of the mucosa, through small molecule transporters (transcellular) or through tight junctions (paracellular), which allow the transfer of molecules smaller than 1.5 nm (20).

Besides, the anatomy of the GI tract shows remarkable flexibility to gut microbial challenges in adults. The interaction of gut microbiota with innate immune cells and pattern recognition receptors regulates cellular and morphologic properties of the GI tract, including the renewal and differentiation of the epithelial lineage, the adaptation of the intestinal microvasculature, and the shape of the ENS and the intestinal smooth muscle layers (21). The ENS is an intrinsic neuronal network that harbors various types of nerve cells located along the GI tract, which not only controls GI motility, fluid homeostasis, and blood flow but also interacts with epithelial and immune cells in the intestine (13). GI motility depends on intrinsic neural and myogenic mechanisms that cooperate with extrinsic neural and hormonal influences (22), which are also largely regulated by gut microbiota. Recent studies also indicate that gut microbiota may be critical for the ENS development and maturation, which is beyond the scope of this review and has been excellently discussed by other review articles (13, 23).

## Gastrointestinal motility is highly dependent on gut microbiota

Gastrointestinal motility is generated by coordination of contraction as well as relaxation of the circular and longitudinal smooth muscles, which is regulated by the ENS, pacemaker cells called ICCs, EECs, and other factors (6, 24, 25). Significant crosstalk between the ENS and EECs modulates GI motility. In response to stimulation, such as microbial metabolite butyrate and other short-chain fatty acids (SCFAs), EECs activate enteric neurons through the release of 5-HT (26). In addition to 5-HT, EECs produce neuropeptides such as somatostatin, motilin, VIP, glucagon-like peptide-1 (GLP-1), and cholecystokinin, which regulate ENS activity in a paracrine manner (27). In addition to these host-specific genetic predispositions, commensal microbiota is also an important modulator of GI motility (7, 28). The common methods used to determine GI motility in animal models *in vivo* include the Evans Blue dye or charcoal propulsion test (small intestinal transit and whole GI transit analysis), bead expulsion test (colonic transit analysis), and fecal pellets collection (defecation frequency) (29). Different methods are used to assess the motility of different intestinal segments, but small intestinal transit analysis involves putting the animals to death, whole GI transit analysis is time-consuming, bead expulsion test needs to be repeated several times, and fecal pellets collection is affected by the environment. A combination of different methods is needed to fully evaluate the GI motility.

Germ-free (GF) and antibiotic-treated animals with gut microbiota deficiency have been used to investigate the role of gut microbiota in GI motility. GF mice have a reduced number of nitrergic neurons and a significant delay in GI motility (30). GF rats display a significant delay in the intestinal transit and the contractility of the small intestine compared to their conventional controls, which is partially reversed by colonization with *Lactobacillus acidophilus* and *Bifidobacterium bifidum* (31). GF mice have lower excitability in the myenteric intrinsic afferent primary neurons (IPANs), which is normalized when GF mice are conventionalized with intestinal bacteria (32). Similarly, mice treated with antibiotics have significantly lower defecation frequency, prolonged intestinal transit time, and loss of enteric neurons in both submucosal and myenteric plexuses in the ileum and proximal colon (33–35). However, the antibiotic application does not completely deplete gut microbiota and may select resistant bacteria or promote fungal outgrowth (36, 37).

**FIGURE 2:**
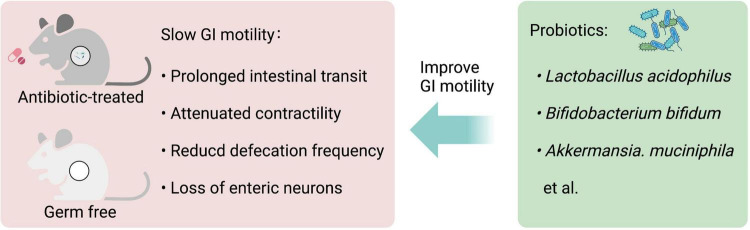
Gastrointestinal (GI) motility is highly dependent on gut microbiota. Both antibiotic-treated and germ-free (GF) rodents that lack gut microbiota have slowed GI motility, with prolonged intestinal transit, attenuated contractility, reduced defecation frequency, and loss of enteric neurons. Probiotic supplements, such as *Lactobacillus acidophilus*, *Bifidobacterium bifidum*, and *Akkermansia. muciniphila* can improve GI motility.

Besides, probiotic supplements improve GI motility in animal and human studies. Daily single administration of *Lactobacillus rhamnosus* GG (LGG) for at least 1 week significantly increases defecation frequency and reduces whole GI transit time in conventional mice in absence of diarrheal phenotype, and the contractions of ileal circular muscle strips of LGG-treated mice show a significant increase in *ex vivo* experiments (38). A combination of four probiotics, *Lactobacillus plantarum 2362*, *Lactobacillus casei ssp. paracase 19*, *Leuconostoc raffinolactis 23*∼*77:1*, and *Pediococcus pentosaceus 16:1*, improves GI motility and protects ICCs in mice with traumatic brain injury (39). *Clostridium butyricum* (*C. butyricum*) suspension promotes ICCs proliferation and improves GI motility (40). Both *Akkermansia. muciniphila* and its outer membrane protein Amuc_1100 improve the GI motility in antibiotic-treated mice (41), indicating some ingredients of dead probiotics may also influence GI motility. Altogether, these studies demonstrate that gut microbiota-derived signals play an important role in the control of GI motility (Figure 2).

Moreover, the altered composition of gut microbiota in the lumen and mucus layer of the GI tract is often accompanied by GI disorders (42). It has been revealed that complex interactions of gut microbiota and host, such as immune and metabolic responses, are involved in the pathophysiology of GI dysmotility. Previous studies show that IBS patients have significant changes in the composition of fecal microbiota, and different subtypes of IBS are associated with different microbiota (43). Intestinal microbiota signatures associated with the severity of IBS symptoms have been identified (44). A major dysbiosis of gut microbiota is observed in constipated-IBS patients, which in turn may influences GI motility and contributes to constipated-IBS pathogenesis (45). Moreover, utilizing a well-defined donor with a specific favorable microbial signature, fecal microbiota transplant (FMT) is an effective treatment for IBS patients, and the response to FMT increases in a dose-dependent manner (46). In contrast, FMT derived from constipated donors delays GI transit time in mice (47). In clinical practice, FMT improves the symptoms of slow transit constipation patients by modulating gut microbiota and metabolites involved in the protein digestion and absorption pathways (48). These data suggest that regulating the gut microbiota may be a novel therapeutic strategy for GI dysmotility.

Gastrointestinal motility disorder is also a complication of GI surgery and various diseases, such as inflammatory bowel disease (IBD) and Parkinson’s disease (PD). GI dysmotility after surgery with reconstruction of the GI tract is common, ranging from POI to malabsorption associated with increased GI motility, in which altered gut microbiota contributes to changes in GI motility (49). Perioperative probiotic supplementation with Bifidobacteria or Lactobacilli improves post-operative recovery in patients undergoing GI surgery (50, 51). IBD shares similar symptoms and some pathophysiology with FGID, such as changes in gut motility associated with inflammatory conditions (52). Gut microbiota dysbiosis has also been demonstrated in IBD, and manipulating gut microbiota with antibiotics, prebiotics, probiotics, or FMT is a promising approach for the treatment of IBD (53). In non-GI diseases, GI dysfunction in PD has been identified and changes in motility play an important role in the GI manifestation of PD (54). Alterations of the gut microbiota in PD have been reported by a large number of studies and dysbiosis may contribute to both the genesis of PD itself and GI complications, such as GI motility disorders (55). Alteration in GI motility and gut microbiota, along with other factors such as diet and drugs, interplay and impact the treatment response in PD patients (54).

## Gut bacteria regulate gastrointestinal motility through their cell wall components and metabolic products

Gut microbiota can directly influence the GI motility through bacterial cell wall components [lipopeptides, peptidoglycan, and lipopolysaccharides (LPS)] binding to TLRs expressed in the GI tract. Indirectly, gut microbiota can also modulate the GI motility *via* the release of metabolites or end products of bacterial biotransformation and fermentation. Three main groups of bacterial metabolites, including BAs, SCFAs, and tryptophan metabolites, have been well studied in the regulation of GI motility. In addition, other microbial metabolites belonging to a wide range of chemical groups have also been shown to modulate GI motility, and there are many more gut microbiota-derived metabolites that need to be identified and investigated for their potential role in the regulation of GI motility (Table 1).

**TABLE 1:** Major bacterial components and metabolites and their effect on gastrointestinal (GI) motility.

Major bacterial components and metabolites	Effects on gastrointestinal (GI) motility
Lipopeptides and peptidoglycan	Signaling through toll-like receptor 2 (TLR2), maintaining adult enteric nervous system and nitrergic neurons
Lipopolysaccharides (LPS)	Signaling *via* TLR4, playing dual function of improving and delaying motility in different manners
Deoxycholic acid (DCA) and lithocholic acid (LCA)	By activating Taketa G-protein-coupled receptor 5 (TGR5), modulating the release of 5-hydroxytryptamine (5-HT) and promoting GI motility
7-ketodeoxycholic acid and muricholic acid	Associated with faster GI transit
Short-chain fatty acids (SCFAs)	Stimulation of glucagon-like peptide-1 (GLP-1) and peptide YY (PYY) production, modulating the release of 5-HT, playing dual function of increasing and decreasing GI motility
Tryptamine	By activating epithelial 5-HT4, accelerating GI transit
Indole-3-carboxaldehyde (IAld)	Activating cholinergic enteric neurons to promote GI motility
5-hydroxytryptophan (5-HTP)	Converted to 5-hydroxyindole (5-HI) by bacterial tryptophanase, improving GI motility directly through activation of L-type voltage-dependent calcium channels (L-VDCCs)
Quercetin	Promoting GI motility and mucin secretion
Putrescine and cadaverine	Regulating intestinal peristalsis
γ-aminobutyric acid (GABA)	Modulating both motor and secretory GI activity

### Gut microbial components regulate gastrointestinal motility *via* binding to toll-like receptors

**FIGURE 3:**
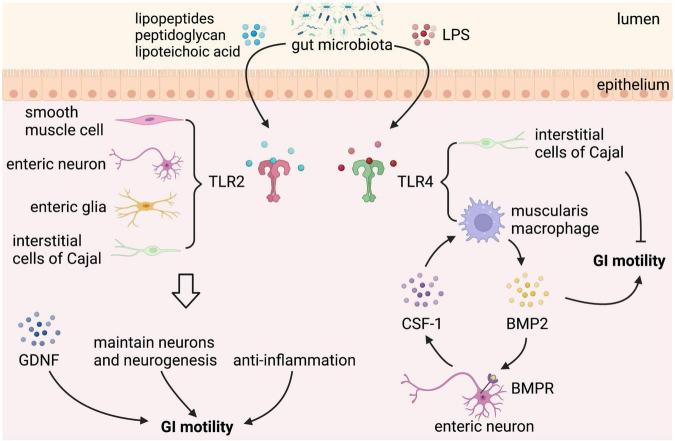
Gut microbial components regulate gastrointestinal (GI) motility *via* binding to toll-like receptor 2/4. Toll-like receptors (TLRs) expressed in the GI tract sense gut microbial components take part in the regulation of GI motility. TLR2 is expressed on enteric smooth muscle cells, neurons, neuroglia, and interstitial cells of Cajal (ICCs). Lipopeptides, peptidoglycan, and lipoteichoic acid from gut microbiota binding to TLR2 stimulate the release of glial cell line-derived neurotrophic factor (GDNF), maintain neurons and neurogenesis, and play an anti-inflammation effect, which can improve GI motility. In addition to TLR2, TLR4 is the best-characterized receptor recognizing gut microbiota-derived Lipopolysaccharide (LPS). LPS binding to TLR4 expressed on muscularis macrophage (MM) stimulates the release of bone morphogenetic protein 2 (BMP2), which improves GI motility. In response to BMP2, enteric neurons produce colony stimulatory factor 1 (CSF-1), which in turn promotes MM homeostasis. However, LPS binding to TLR4 expressed on ICCs has a negative effect on GI motility.

Gut microbiota can directly affect the GI motility, which is mediated by bacterial cell wall components, such as lipopeptides, peptidoglycan, and LPS, through binding to certain subtypes of the host toll-like receptors (TLRs), which are expressed in intestinal epithelial cells, neurons, neuroglia, and smooth muscle cells (56–59). TLRs can directly interact with bacterial components to facilitate communication between gut microbiota and GI cells. However, given the fact that TLRs are expressed by several types of cells in the GI tract, it is difficult to identify the specific cells connecting the signaling between gut microbial components and the GI motility. Among all the TLRs, TLR2 and TLR4 are the most important bacteria-sensing receptors that regulate the ENS and gut motility (Figure 3). TLR2 recognizes lipopeptides and peptidoglycan, whereas TLR4 recognizes LPS (60).

TLR2 is expressed on enteric smooth muscle cells, neurons, and neuroglia of the intestinal tract. TLR2 knockout mice have reduced βIII-tubulin+ neurons and fibers in submucosal plexus with a lower number of HuC/D+ neurons, S100β+ enteric glial cells (EGC), and neuronal nitric oxide synthase positive (nNOS+) neurons in myenteric plexus, which is accompanied by a loss of nitrergic modulation in intestinal contractility (58). Besides, a reduced glial-cell-line derived neurotrophic factor (GDNF) signaling is found in TLR2 knockout mice, and TLR2 agonists upregulate GDNF expression of isolated longitudinal smooth muscle-myenteric plexus (LMMP) *via* nuclear factor-κB (NF-κB) and p38 mitogen-activated protein kinase (MAPK) signaling. GDNF administration ameliorates ENS defects and GI dysmotility in TLR2 knockout mice and wild-type (WT) mice treated with antibiotics, confirming that the gut microbiota-TLR2-GDNF axis plays an important role in ENS and GI motility (58). In addition, antibiotic-treated mice show reduced expression of TLR2 in the ileum and colon (61), with a delay of GI transit, a significantly reduced frequency of stool expulsion, loss of myenteric plexus neurons (reduced number of AChE+ and nNOS+ myenteric plexus neurons and a proportional increase of SP+ myenteric plexus neurons), which are partly restored by activation of TLR2 signaling (62).

By activating TLR2 expressed in smooth muscle, lipopeptides, the main components of the intestinal gram-positive bacteria, may possess anti-inflammatory properties that can restore GI motor function in a MyD88-independent manner (63). Mice given a TLR2 antagonist have significant dysmotility with prolonged whole gut transit times (WGTT) and lipoteichoic acid (LTA), a bacteria-derived TLR2 agonist, counteracts the prolonged effect of ampicillin on WGTT, and the underlying mechanism is that gut microbiota-regulated specific TLR2 signaling processes help to maintain nitrergic neurons and neurogenesis in the intestine (59).

Other specific cell wall components of commensal bacteria can also directly interact with TLR2. Amuc_1100, an outer membrane protein of *Akkermansia muciniphila* (*A. mucinphila*), promotes the intestinal biosynthesis of serotonin (5-HT) and further improves the function of GI motility through TLR2 signaling (41). *Clostridium butyricum* (*C. butyricum*), a probiotic strain, increase the secretion of ghrelin and SP and may promote GI motility by inducing the cell viability of ICCs *via* activation of NF-κB and JNK in a TLR2-dependent manner, but what bacterial components take the effect has not been identified (40). Besides, *Bacteroides thetaiotaomicron* (Bt), a human resident gut microbe, is able to increase colonic motor complexes and restore the downregulated TLR2 expression in the colon of GF mice (64), suggesting that Bt is likely to regulate GI motility *via* TLR2 expression.

In addition to TLR2, TLR4 is the best-characterized receptor recognizing gut microbiota-derived LPS, a major membrane component of gram-negative bacteria, and also plays an important role in the regulation of ENS and GI motility. TLR4 knockout mice exhibit reduced defecation frequency, delayed colonic transit, impaired nitrergic colonic relaxation, and loss of nNOS+ neurons, leading to intestinal dysmotility (30). A similar phenotype was also observed in LPS-hyporesponsive *C3H/HeJ* mice and enteric neuronal-specific Myd88, a key adaptor signaling molecule for TLRs, knockdown mice (30). Furthermore, gliosis in ileal myenteric plexus and a reduced cholinergic excitatory response are found in TLR4 knockout mice, which depend on enhanced inhibitory neurotransmission mediated by both NO and ATP through nitrergic and purinergic pathways (65), indicating that TLR4 signaling is essential for proper bidirectional communication between neuron and glia in the regulation of GI motility.

Besides, low-dose LPS treatment improves the survival of primary enteric neurons isolated from WT mice but not from LPS-hyporesponsive mice in an NF-*k*B-dependent manner (30). *In vivo*, LPS supplementation partially improves GI motility in antibiotic-treated mice by modulating the intestinal mucosal immune system (17). The intestinal immune system plays an important role in maintaining the homeostasis of the gut. MMs, a subtype of macrophages that reside in close contact with the myenteric plexus in the muscularis mucosa, secrete bone morphogenetic protein 2 (BMP2) in response to stimuli from commensal microbiota, like LPS. BMP2 regulates GI motility at a stable state by activating the BMP receptor (BMPR) expressed on the enteric neurons, and in response to BMP2, enteric neurons produce colony stimulatory factor 1 (CSF1), which in turn promotes MM homeostasis (17). Thus, plastic crosstalk between MMs and enteric neurons is driven by gut microbiota that controls GI motility. However, in a recent study, LPS supplementation at the same concentration only prevents antibiotic-induced neuronal loss but does not reverse or attenuate antibiotic-induced alterations in GI function (35). On the contrary, higher does LPS has an inhibitory effect on GI motility. The inflammation response of the GI tract induced by LPS results in smooth muscle dysfunction and resultant GI paralysis (66). Through binding to TLR4, LPS can inhibit the pacemaker currents in ICCs through NF-κB and p38 MAPK signaling pathway *via* prostaglandin E2- and NO-dependent mechanism (67). Time-dependent changes are also observed in the inhibitory action of LPS on GI motility. In the early phase of LPS exposure, LPS induces cyclooxygenase-2 (COX-2) to produce PGE2, which inhibits contractility *via* activating PGE2 receptors on smooth muscle cells, and in the late phase, iNOS is induced to produce NO, which in turn inhibits contraction (68). Therefore, both increased and decreased GI motility has been reported when the gut is exposed to LPS, which may be due to the dose and type of LPS, the region of the GI system that is studied, and the timing of motility assessment.

### Different gut microbial metabolites regulate gastrointestinal motility in different ways

#### Bile acids

Bile acids are produced from cholesterol in the liver, stored in the gallbladder, and released into the GI tract upon food intake. In the GI tract, conjugated primary BAs can be deconjugated to unconjugated BAs and further dehydroxylated to secondary BAs by the gut microbiota (69). In addition to their role in the normal digestion and absorption of dietary fat, tryptic cleavage of dietary proteins, and antimicrobial effects, BAs take part in secretion and GI motility, and abnormal delivery of BAs to the intestine caused by disease or therapy results in GI disorders, such as constipation and diarrhea (70, 71). Luminal BAs have region-specific effects on GI motility. They inhibit the small intestine motility, which may slow ileal transit and contribute to efficient absorption (72, 73). In contrast, BAs promote large intestine motility (74). Patients with constipation-predominant IBS have a lower level of total BAs in feces compared to healthy controls (75). An increased proportion of fecal primary BAs is observed in diarrhea-predominant IBS, which may be due to the altered composition of gut microbiota (76), given that gut microbiota has an exclusive role in the transformation of primary BAs into secondary BAs.

Deoxycholic acid (DCA) and lithocholic acid (LCA), the major secondary BAs produced by microbial biotransformation in the colon, are the most efficient agonists of Taketa G-protein-coupled receptor 5 (TGR5) (77), which have been reported to improve GI motility. TGR5 is a plasma membrane BA receptor and is expressed by the EECs and enteric neurons in the intestine. DCA has been shown to activate TGR5 on EECs to stimulate the release of 5-HT, a major regulator of GI secretion and motility, and GLP-1, an incretin and mediator of the ileal brake (78, 79). DCA promotes peristaltic contractions of the colon by stimulating a concentration-dependent release of 5-HT and calcitonin gene-related peptide (CGRP), the major neurotransmitters of the afferent limb of the peristaltic reflex, *via* activating TGR5 expressed on colonic ECs and IPANs, respectively (29). TGR5 deficiency leads to slower GI transit and reduced frequency of defecation and fecal water content whereas TGR5 overexpression accelerates colonic transit in mice (29). Thus, TGR5 is a key mediator that gut microbiota-dependent production of secondary BAs influences GI motility.

Particular *Clostridium* species possess high 7α-dehydroxylation activity required for the production of DCA from cholic acid (80, 81). Indigenous spore-forming microbes (Sp), comprised largely of Clostridia, colonization ameliorates GF-associated GI dysmotility with reduced total transit time, increased fecal output, and increased colonic activation of IPANs in the myenteric plexus (82). This may be mediated by Sp-induced colonic 5-HT biosynthesis, given that Sp colonization of GF mice completely restores serum and colon 5-HT, elevates host colonic expression of tryptophan hydroxylase (TPH1, a rate-limiting enzyme in the 5-HT synthesis pathway in ECs), and decreases expression of SLC6A4 to levels observed in SPF mice (82). Besides, 5-HT modulated by gut microbiota is associated with neurogenesis in the ENS and intestinal transit, potentially *via* the 5-HT4 receptor (83). Specific microbial factors that may be responsible for the serotonergic effects of Sp have been identified. Sp-induced increases in DCA and other microbial metabolites likely contribute to its ability to improve GI motility by promoting Tph1 expression and 5-HT biosynthesis in colonic ECs (82).

**FIGURE 4:**
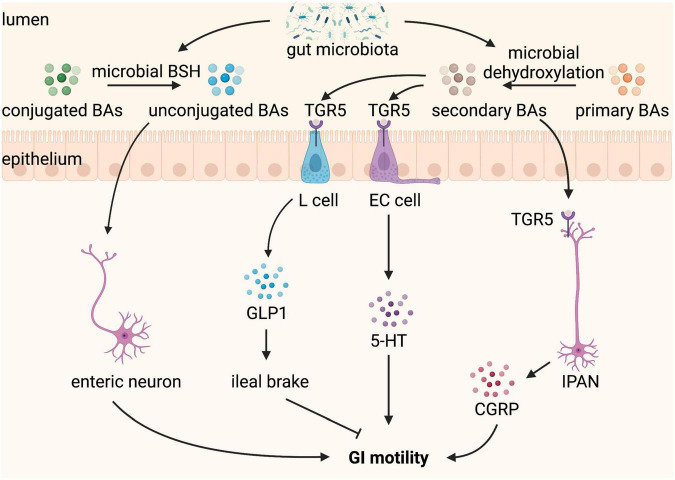
Bile acids (BAs) converted by gut microbiota regulate gastrointestinal (GI) motility. BAs are produced from cholesterol in the liver and released into the GI tract when food intake. In the GI tract, conjugated BAs can be converted by microbial bile salt hydrolase (BSH) to unconjugated BAs, which stimulate enteric neurons and promote GI motility. Primary BAs can be converted by microbial dehydroxylation to secondary BAs, which binding to Taketa G-protein-coupled receptor 5 (TGR5) expressed L cell and enterochromaffin (EC) cell stimulate the release of glucagon-like peptide 1 (GLP-1) and 5-hydroxytryptamine (5-HT), respectively. GLP1 leads to ileal brake and slows GI motility, whereas 5-HT promotes GI motility. TGR5 is also expressed on intrinsic afferent primary neurons (IPANs), which can be activated by secondary BAs and produce calcitonin gene-related peptide (CGRP) improving GI motility.

In addition, unconjugated BAs are also reported to regulate gut sensorimotor activity (84). Unconjugated BAs, including 7-ketodeoxycholic acid and muricholic acid, produced by microbial bile salt hydrolase (BSH) are correlated with faster transit time and affect GI motility *via* modulation of Ret signaling in the ENS (85). GI transit time is significantly decreased in mice colonized with BSH-positive microbiota, indicating that GI motility is dependent on gut microbiota-mediated deconjugation of BAs (85). Moreover, greater BSH activity of gut bacteria drives faster colonic transit, with greater prokinetic effects in males than in females (86). In summary, gut microbiota indirectly regulates GI motility through its effect on the modification of BAs composition by deconjugation and dehydroxylation (Figure 4).

#### Short-chain fatty acids

Short-chain fatty acids, a major class of bacterial metabolites, can directly activate G protein-coupled receptors, inhibit histone deacetylases, and serve as energy substrates and thus play a key role in the regulation of host physiology, including gut motility (87). SCFAs, including acetate, propionate, and butyrate, are produced by gut microbial fermentation of dietary indigestible polysaccharides (87). A recent meta-analysis shows that constipation-predominant IBS (IBS-C) patients have decreased concentrations of fecal propionate and butyrate, whereas diarrhea-predominant IBS (IBS-D) patients have increased concentrations of butyrate compared to healthy controls, indicating the significant role of SCFAs in regulating GI motility (88).

Germ-free and antibiotic-treated mice have reduced SCFAs, increased proglucagon (*Gcg*) expression in L cells of the colon, and increased GLP-1 in the plasma (89). GLP-1 as an enterogastrone affects the regulation of gastric emptying and GI transit (90). Overexpression of GLP-1 is associated with markedly prolonged GI transit in patients with neuroendocrine tumors (91), and elevated GLP-1 and slower GI transit have also been found in patients with anorexia nervosa (92). SCFAs, particularly butyrate, are a primary unique energy source for colonocytes (93). Increasing energy availability by supplementing SCFAs or SCFAs-producing bacteria suppresses Gcg expression in the colon of GF mice, suggesting that colonic L cells sense local energy availability and regulate basal GLP-1 secretion (89). Colonic-derived GLP-1 has an important function in slowing GI motility in order to allow more time for nutrient absorption when energy availability is insufficient (89).

Butyrate also directly regulates the ENS and controls GI motility involving at least in part the monocarboxylate transporter 2 (MCT2) of enteric neurons (94). MCTs, proton-linked membrane proteins of the SLC16A family, are responsible for the transport of butyrate into cells (95). Butyrate-induced ChAT expression involves the acetylation of histone H3 lysine 9 (H3K9) and the Src-kinase signaling pathway, which increases cholinergic phenotype resulting in increased colonic contractility and shorter colonic transit time (94). Administration of acetate promotes GI motility through regulating 5-HT synthesis, neurotrophic factors expression, and immunocyte differentiation by HDAC3 inhibition in the colon (96). All three SCFAs promote colonic peristalsis by stimulating 5-HT release, which activates 5-HT4 receptors located on intrinsic CGRP-containing sensory neurons (97). However, it also has been reported that different SCFAs exert different effects on proximal and distal colonic motility in guinea pigs, in contrast to butyrate, acetate and propionate seem to decrease colonic motility, suggesting that the net effects of SCFAs depend on the balance of SCFAs produced by gut microbiota (98). Luminal propionate reduces anion secretion and slows colonic motility *via* PYY mediation and enteric sensory neuron activation, by stimulating GPR43 and GPR41, respectively (99). The difference in the effects of different SCFAs may be due to the different SCFA concentrations and animal models used in the study because it has been reported that acetate and butyrate can significantly affect *TPH1* mRNA expression of ECs in a concentration-dependent manner *in vitro* (100). Moreover, chronically elevated SCFAs lead to a disbalance of activating and inhibiting action resulting in a detrimental increased colonic transit rate, which may play a role in the pathogenesis of diarrhea-predominant IBS (101).

**FIGURE 5:**
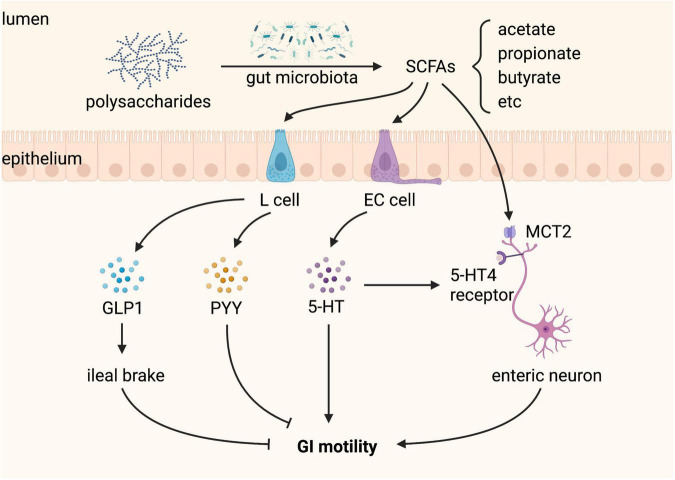
Short-chain fatty acids (SCFAs) produced by gut microbiota regulate gastrointestinal (GI) motility. SCFAs, including acetate, propionate, and butyrate, are produced from gut microbial fermentation of dietary polysaccharides. L cells sense SCFAs and produce glucagon-like peptide 1 (GLP-1) and peptide YY (PYY), both of which inhibit GI motility. Enterochromaffin (EC) cells sense SCFAs and produce 5-hydroxytryptamine (5-HT), which promotes GI motility by activating the 5-HT4 receptor expressed on enteric neurons. SCFAs can also stimulate enteric neurons through monocarboxylate transporter 2 (MCT2), playing a positive role in GI motility.

In addition, SCFAs alone enhance the survival of enteric neurons and promote enteric neurogenesis in antibiotic-treated mice, but do not affect GI function (35). Why the beneficial effects of SCFAs in the ENS structure of antibiotic-treated mice are not accompanied by recovery of GI function remains to be elucidated. Nevertheless, SCFAs produced by gut microbiota regulate GI motility (Figure 5).

#### Tryptophan metabolites

Tryptophan (Trp) metabolism has appeared as a crucial metabolic pathway involved in the host-microbiota crosstalk, which plays a central role in maintaining GI function (102). Trp is an essential amino acid that entirely depends on dietary intake, and its metabolism in the gut follows three major pathways: (1) the indole pathway: direct transformation by commensal microbiota into indole and indole derivatives; (2) the kynurenine pathway: metabolized by epithelial and immune cells into kynurenine through indoleamine 2,3-dioxygenase 1 (IDO1); and (3) the serotonin pathway: conversion by ECs into 5-HT through TpH1, all of these pathways are directly or indirectly controlled by gut microbiota (103). In various GI diseases, Trp metabolic disorders have been found at least one of these pathways, as in inflammatory bowel diseases (IBD) and celiac disease (104, 105), hence it is necessary to expound the role of gut microbiota-controlled tryptophan metabolism in GI motility.

Tryptamine is a by-product of the indole pathway of Trp metabolism by the gut microbiota and is abundant in human and rodent feces. Tryptamine increases cAMP release from the epithelium and anion-dependent fluid secretion in the proximal colon, which is mediated by activating colonic epithelial GCPR 5-HT4R alone, not 5-HT4Rs expressed in colonic mucosa and neuronal plexus (106). Engineered *Bacteroides thetaiotaomicron* optimized to express tryptophan decarboxylase, the enzyme responsible for decarboxylation of tryptophan to tryptamine, effectively colonizes the gut, which produces tryptamine *in vivo* and can accelerate WGTT by increasing colonic secretion (106). Accordingly, a longitudinal multi-omics study in humans demonstrates that tryptamine is elevated in the stools of a subset of IBS patients with diarrhea (107). In addition, tryptamine, as an aryl hydrocarbon receptor (AhR) ligand (103), may also regulate GI transit through other different mechanisms. Gut microbiota-dependent AhR expression and activation in neurons of the distal GI tract enables these neurons to respond to the luminal environment, thereby regulating intestinal peristalsis, but the detailed molecular mechanism underlying the downstream pathway of neuronal AhR signaling remains to be characterized (34). Other bacteria-derived tryptophan metabolites produced from the indole pathway, including indole and indole-3-carboxaldehyde (IAld), are also AhR agonists, but they can promote GI motility in an AhR-independent manner by activating EECs, through transient receptor potential ankyrin A1 (Trpa1), increase neurotransmitter 5-HT secretion by ECs, and stimulate IPANs, which then activate cholinergic enteric neurons to promote GI motility (108). 5-hydroxytryptophan (5-HTP) is a chemical precursor and intermediate metabolite of Trp in the biosynthesis of 5-HT and is often used as a food supplement or as a drug. Administration of 5-HTP restores 5-HT to the ENS and normalizes GI motility and growth of the enteric epithelium in a mouse model of depression (109). 5-HTP can be converted to 5-hydroxyindole (5-HI) by bacterial tryptophanase, which is dependent on the gut microbiota composition and pH levels. 5-HI improves GI motility directly through activation of L-type voltage-dependent calcium channels (L-VDCCs) located on the colonic smooth muscle cells and possible *via* its induction of 5-HT release from ECCs activating 5-HT3 and 5-HT4 receptors on afferent nerve terminals from the ENS (110).

**FIGURE 6:**
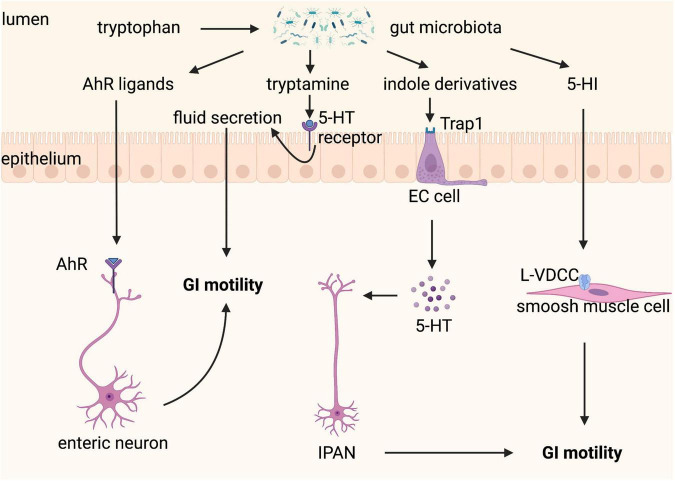
Tryptophan metabolism controlled by gut microbiota regulates gastrointestinal (GI) motility. Tryptophan can be metabolized by gut microbiota to a variety of active substances. Aryl hydrocarbon receptor (AhR) ligands binding to AhR expressed on enteric neurons promote GI motility. Tryptamine contributes to fluid secretion by activating the 5-hydroxytryptamine (5-HT) receptor on enterocytes, which increases GI motility. Indole derivatives stimulate the release of 5-HT from enterochromaffin (EC) cells *via* transient receptor potential ankyrin A1 (Trpa1), which improves GI motility through stimulating intrinsic afferent primary neurons (IPANs). 5-hydroxyindole (5-HI) can directly act on smooth muscle cells *via* L-type voltage-dependent calcium channels (L-VDCCs) and then promotes GI motility.

These studies confirm that gut microbiota-controlled Trp metabolism is a crucial factor in the crosstalk of host-microbiota and the fine-tuning of GI motility (Figure 6), indicating that Trp metabolism is a potential therapeutic target for GI motility disorders. However, it is a complex therapeutic target because modulation of one of its three metabolic pathways will affect the others, therefore consequences for all of its three metabolic pathways should be considered when modifying gut microbiota and Trp metabolism.

#### Other gut microbial metabolites

Other products of gut microbial metabolization of nutrients also have an effect on the regulation of GI motility. Quercetin is an abundant flavonoid in many vegetables, fruits, and grains (111). It is also produced by gut bacteria, specifically Fusobacteria species (112), and promote GI motility and mucin secretion in rat with loperamide-induced constipation through regulation of the muscarinic acetylcholine receptor (mAChR) signaling pathway (113). Putrescine and cadaverine, which are polyamines and trace amine, respectively, produced by gut bacteria may act on chemosensors and thus regulate intestinal peristalsis in rate (114). Besides, gut microbiota mediates the production of neurotransmitters, such as γ-aminobutyric acid (GABA) produced from glutamic acid, involved in gut motility (115, 116). GABA receptors are expressed in the GI tract, where GABA modulates both motor and secretory GI activity (117). The dietary histidine can be metabolized by *Morganella morganii* and *Lactobacillus reuteri* into histamine, which shapes colonic motility through activating histamine receptors along the GI tract (118). Except for SCFAs, saturated long-chain fatty acids, such as heptadecanoic acid and stearic acid, produced by gut bacteria can promote colonic muscle contraction and increase stool frequency in rats (119). Altogether, these metabolites produced by gut microbiota play a crucial role in the regulation of GI motility.

## Conclusion

Mechanisms of GI motility are complex. Previous studies support the notion of close crosstalk between the host and gut microbiota, involving multiple integrated gut microbiota-controlled signaling pathways on the host to modulate GI motility. Taken together, a variety of gut microbiota-derived signals are orchestrated and cooperate with each other in the modulation of GI motility. Gut microbial components and metabolites appear to have multiple effects on GI motility. A comprehensive understanding of these roles of gut microbiota-derived signals in GI motility will enable the further development of rational specific therapies to either directly prevent or improve GI dysmotility. However, the role of gut microbiota-derived signals in GI motility discussed in this review is mostly based on animal experiments, lessons learned from animal models still need to be confirmed in clinical settings. This process will elucidate gut microbiota-dependent mechanisms that modulate GI motility and facilitate the development of gut microbiota-targeted therapeutic approaches to improve GI diseases with dysmotility.

## Author contributions

ZZ managed the major literature research and wrote the first draft of the manuscript. JT and YH managed literature research and reviewed the manuscript. WZ critically reviewed the manuscript and provided valuable discussions and criticisms. All authors contributed to the article and approved the submitted version.

## Conflict of interest

The authors declare that the research was conducted in the absence of any commercial or financial relationships that could be construed as a potential conflict of interest.

## Publisher’s note

All claims expressed in this article are solely those of the authors and do not necessarily represent those of their affiliated organizations, or those of the publisher, the editors and the reviewers. Any product that may be evaluated in this article, or claim that may be made by its manufacturer, is not guaranteed or endorsed by the publisher.
